# Colo-salpingeal fistula: A rare case report

**DOI:** 10.1016/j.radcr.2024.08.049

**Published:** 2024-09-07

**Authors:** Bahman Rasuli

**Affiliations:** Advanced Diagnostic and Interventional Radiology Research Center (ADIR), Imam Khomeini Hospital Complex, Tehran University of Medical Sciences, Tehran, Iran

**Keywords:** Fallopian tube, Salpingo-enteric fistula, Hysterosalpingogram, Infertility

## Abstract

Salpingo-enteric fistula is a rare condition that can cause infertility. It occurs when there is an abnormal connection between the fallopian tube and the intestine. Accurate diagnosis can be made using hysterosalpingography.The case of a 40-year-old asymptomatic woman diagnosed with primary infertility and scheduled for a hysterosalpingogram as part of the routine workup in the infertility clinic. There was no history of pelvic inflammatory disease or previous surgical intervention. The result showed presence of bilateral hydrosalpinx without peritoneal spillage. Contrast leaked into the adjacent descending colon and sigmoid loop on the left side. Asymptomatic salpingo-enteric fistulas may be a hidden cause of infertility, and their association can only be confirmed if all other factors of infertility have been convincingly ruled out.

## Introduction

Communication between the gastrointestinal tract and the female adnexal structure is rare. There have been very few reported cases of tubo-enteric fistulas in the literature [1]. Considering the proximity of the uterus to surrounding adnexal structures, various types of fistulas may form due to pelvic disease, radiation therapy, or prior intervention [2].

Enterotubal fistula may occur as a result of pelvic inflammatory disease, tuberculosis, Crohn's disease, endometriosis, diverticulitis, appendicitis, postlower segment cesarean section, or after surgery for tuberculosis [3]. Clinical complaints can range from being asymptomatic to experiencing symptoms such as amenorrhea, cyclic hematuria, passage of urine or feces through the vaginal tract, perineal dermatitis, foul-smelling air, or discharge through unknown orifices [4].

Moreover, demonstrating the fistulous tract and identifying its underlying cause is quite laborious and challenging. A variety of radiological modalities are available to identify fistulous communications, but almost all previously documented salpingo-enteric fistula cases were discovered incidentally by hysterosalpingography in infertile women[4]. For this condition, the best approach is surgical management [3].

The purpose of this case report is to present a rare finding on hysterosalpingography, which may be a contributing factor to infertility in asymptomatic women.

## Case presentation

A 40-year-old woman visited the infertility clinic at our Hospital due to being unable to conceive for 1 year after getting married in 2024. She did not have any symptoms and had no history of pelvic inflammatory or bowel diseases. Her family history did not show any diseases, including tuberculosis, and she had not undergone surgical intervention. Her husband was clinically normal, and all investigations for potential causes of infertility were normal.

Physical examination revealed a healthy nulligravida female with no relevant abnormal general or local genital physical findings.

The patient came in for a hysterosalpingogram on the nineth day of her menstrual cycle. A hydrosoluble nonionic contrast medium (Omnipaue 350 mg) was injected into the uterine cavity under an aseptic technique through Leech Wilkinson's cannula.

The uterus was centrally located in the pelvic cavity, displaying a normal shape and contour. The cervical canal demonstrated normal length, mucosal surface, and proper expansion.

**Fig. 1:**
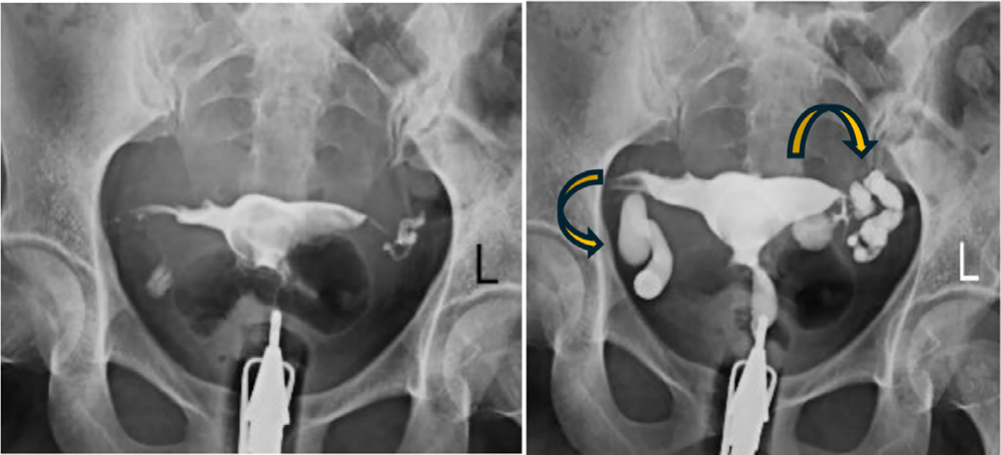
Bilateral distal fallopian tube block with fluid-filled dilatation of the tubes (curved arrows) and without immediate or peritoneal spillage.

Both fallopian tubes were filled and showed abnormal peristalsis and convulsions followed by gradual tubal dilatation and retained contrast material due to hydrosalpinx (Fig. 1). No free intraperitoneal spillage of contrast was seen from fallopian tubes.

**Fig. 2:**
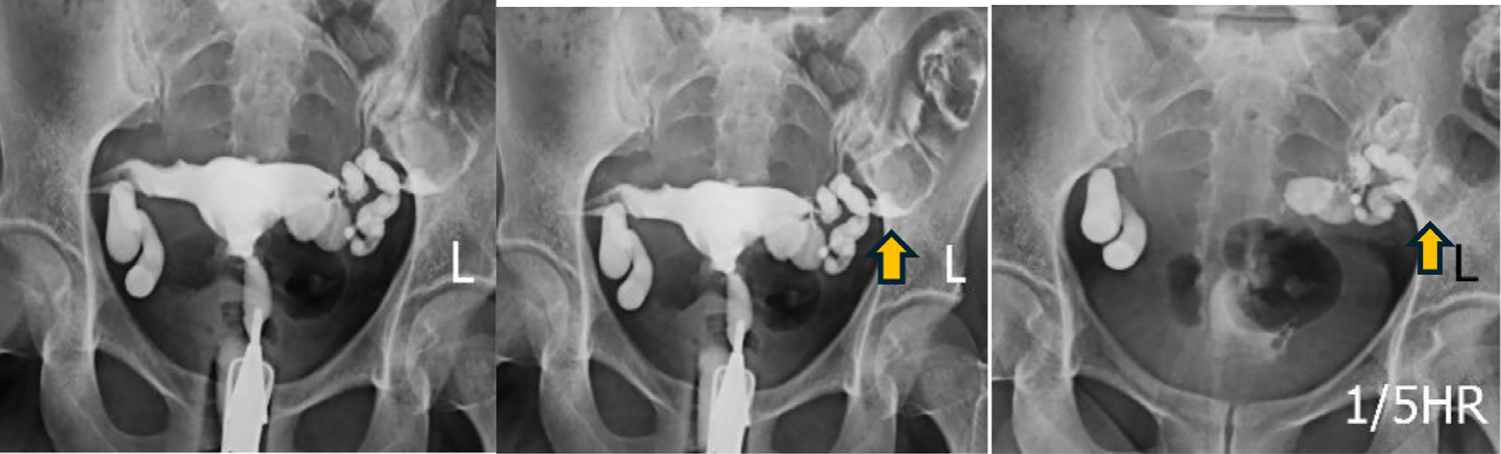
Subsequent HSG films showed contrast opacification of the descending colon and sigmoid loop through the dilated left fallopian tube (arrows).

Contrast from the left fallopian tube was seen outlining the lumen of the distal part of the descending colon and proximal part of the sigmoid loop as evidenced by the presence of haustrations within the opacified bowel on delayed images (Fig. 2). The diagnosis of the colo-salpingeal fistula was made on the left side.

Further evaluations, including a pelvic ultrasound examination to search for any tubo-ovarian mass or abscess, and a chest X-ray to rule out tuberculosis, were performed. The findings were essentially normal.

The patient was given the option to choose between surgical open laparotomy and laparoscopic approach for the repair of the fistula and salpingoplasty. The patient chose to undergo laparoscopy, and the procedure was successful with a combined team of surgeons and obstetricians. The patient remained stable and has been followed up for 6 months post-treatment. She is currently visiting the IVF unit in this hospital for possible assisted conception.

## Discussion

Salpingo-enteric or tubo-enteric fistula is a very rare incidental finding. Although the pathogenesis is not fully understood, it has been suggested to occur as a result of the blockage of the tubal lumen by inflammatory material, leading to the formation of fistulous tracts opening at other sites [3].

Fistulas have been reported between the fallopian tube and the rectum, sigmoid, appendix, cecum, and ileum. The adhesions between the intestinal wall and the fallopian tube may develop during an acute episode of diverticulitis, leading to necrosis and the formation of a fistula. This pathogenesis may clarify the occurrence of a colosalpingeal fistula as a result or Crohn's disease. Neoplastic fistulas can also form through a similar mechanism involving inflammation, epithelial necrosis, and associated ulceration.

Colosalpingeal fistula occurs in the left fallopian tube in 93% of cases, while the right tube is affected in only 7% of cases, due to cecal diverticulosis. So far, there has only been one reported case of bilateral salpingocolic fistula in the medical literature [5].

Patients with a salpingo-enteric fistula may be asymptomatic. When symptoms are present, they are usually related to the underlying disorders, such as pelvic inflammatory disease, inflammatory bowel diseases, diverticular diseases, endometriosis, and tuberculosis [4,6,7]. The diagnosis of a salpingo-enteric fistula is often discovered incidentally during a hysterosalpingogram (HSG), commonly due to infertility issues. This was the finding in the case presented.Typical hysterosalpingography findings include hydrosalpinx, architectural distortion of the fallopian tube, abnormal pelvic location of the fallopian tube accompanied by visualization of contrast outlining the bowel mucosa and then its further transit into distal bowel loops on subsequent films. Occasionally, salpingo-enteric fistulas may be diagnosed during a barium enema examination or laparotomy, especially those resulting from Crohn's disease and complicated diverticulosis [6]. However contrast enema examination is a less sensitive diagnostic test due to the small lumen of the fistula and the failure to generate adequate pressure between the colon and fallopian tube, which prevents contrast filling the tract [3]. It is important to note that we cannot definitively determine the contribution of the salpingo-enteric fistula to infertility in this patient. It can be considered an associated factor that may have contributed to the pelvic inflammatory disease observed in the patient.

Due to a high risk of recurrence, conservative, nonsurgical treatment is ineffective and is only recommended for patients with comorbidities and high anesthesia risk, or for those who decline surgical treatment [5]. Therefore predominant treatment option is surgical, involving fistula closure with salpingectomy as the most effective method to prevent subsequent ectopic pregnancy in this rare disease [2]. En-bloc fistula resection and salpingectomy are also recommended for those resulting from Crohn's disease and complicated diverticulitis; however, the type of resection will be tailored to the peculiarity of the patient [2].

## Conclusion

Salpingoenteric fistulae, a rare and silent disease, can lead to infertility. The use of hysterosalpingography as a complementary modality for investigating infertility is once again strengthened. A hysterosalpingography is typically used to confirm the diagnosis, and assisted reproductive technology can help these patients achieve a successful pregnancy. After surgery, careful postoperative care can help protect the patient from both common and uncommon complications.

## Patient consent

Written informed consent from the patient for publication has been obtained.
